# Magnetically controlled assembly: a new approach to organic integrated photonics

**DOI:** 10.1039/d3sc01779f

**Published:** 2023-07-26

**Authors:** Lixin Xu, Hao Jia, Chuang Zhang, Baipeng Yin, Jiannian Yao

**Affiliations:** a Beijing National Laboratory for Molecular Sciences, Institute of Chemistry, Chinese Academy of Sciences Beijing 100190 China jnyao@iccas.ac.cn yinbaipeng@iccas.ac.cn; b University of Chinese Academy of Sciences Beijing 100049 China

## Abstract

Hierarchical self-assembly of organic molecules or assemblies is of great importance for organic photonics to move from fundamental research to integrated and practical applications. Magnetic fields with the advantages of high controllability, non-contact manipulation, and instantaneous response have emerged as an elegant way to prepare organic hierarchical nanostructures. In this perspective, we outline the development history of organic photonic materials and highlight the importance of organic hierarchical nanostructures for a wide range of applications, including microlasers, optical displays, information encoding, sensing, and beyond. Then, we will discuss recent advances in magnetically controlled assembly for creating organic hierarchical nanostructures, with a particular focus on their potential for enabling the development of integrated photonic devices with unprecedented functionality and performance. Finally, we present several perspectives on the further development of magnetically controlled assembly strategies from the perspective of performance optimization and functional design of organic integrated photonics.

## Abstract 2

This perspective focuses on the development of organic hierarchical nanostructures based on magnetically controlled assembly methods, which are guided by the requirements of organic integrated photonics.

## Graphical abstract

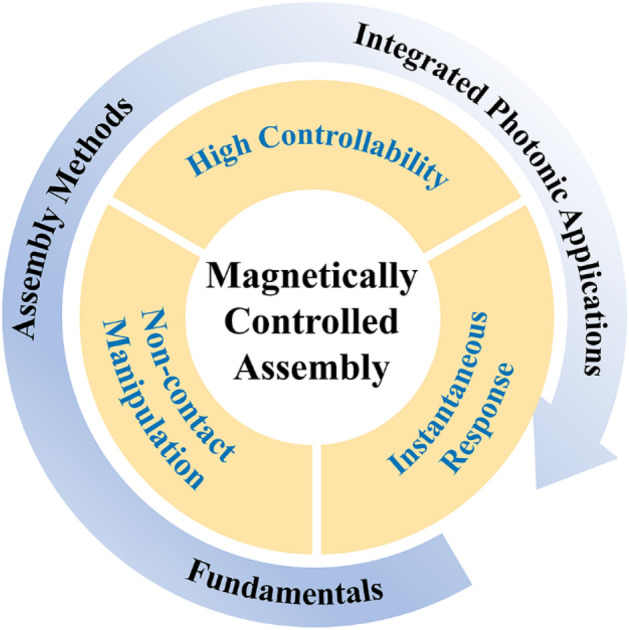

## 1. Introduction

The use of photons instead of electrons as information carriers presents a promising approach to overcome the speed limitations and power consumption issues associated with traditional electronics, which has given rise to photonics.^1–3^ To achieve photonic devices for all-optical signal processing, a viable approach is to create photonic building blocks that function as analogues to electronic circuits, including photonic analogues of power sources,^4,5^ diodes,^6–9^ and transistors.^10,11^ Nevertheless, there are significant differences between photonic analogies and their corresponding electronic components.^12^ The intrinsic characteristics of photons indicate that the flow of light follows time-reversal symmetry, which in turn means that photon interaction is weaker compared to electrons.^13,14^ This poses a significant challenge for the operation of photons, which is important in achieving all-optical signal processing. Fortunately, opto-functional materials can transmit, absorb, store, and convert light, enabling the indirect operation of photons through light–matter interactions.^15–18^ Therefore, exploring advanced opto-functional materials has become a crucial factor in the development of photonic integrated circuits. Among them, organic semiconductors are of current interest in photonics due to their adjustable optoelectronic properties,^19,20^ high optical gain^21,22^ excellent compatibility,^23,24^ and cost-effectiveness.^25^

**Fig. 1:**
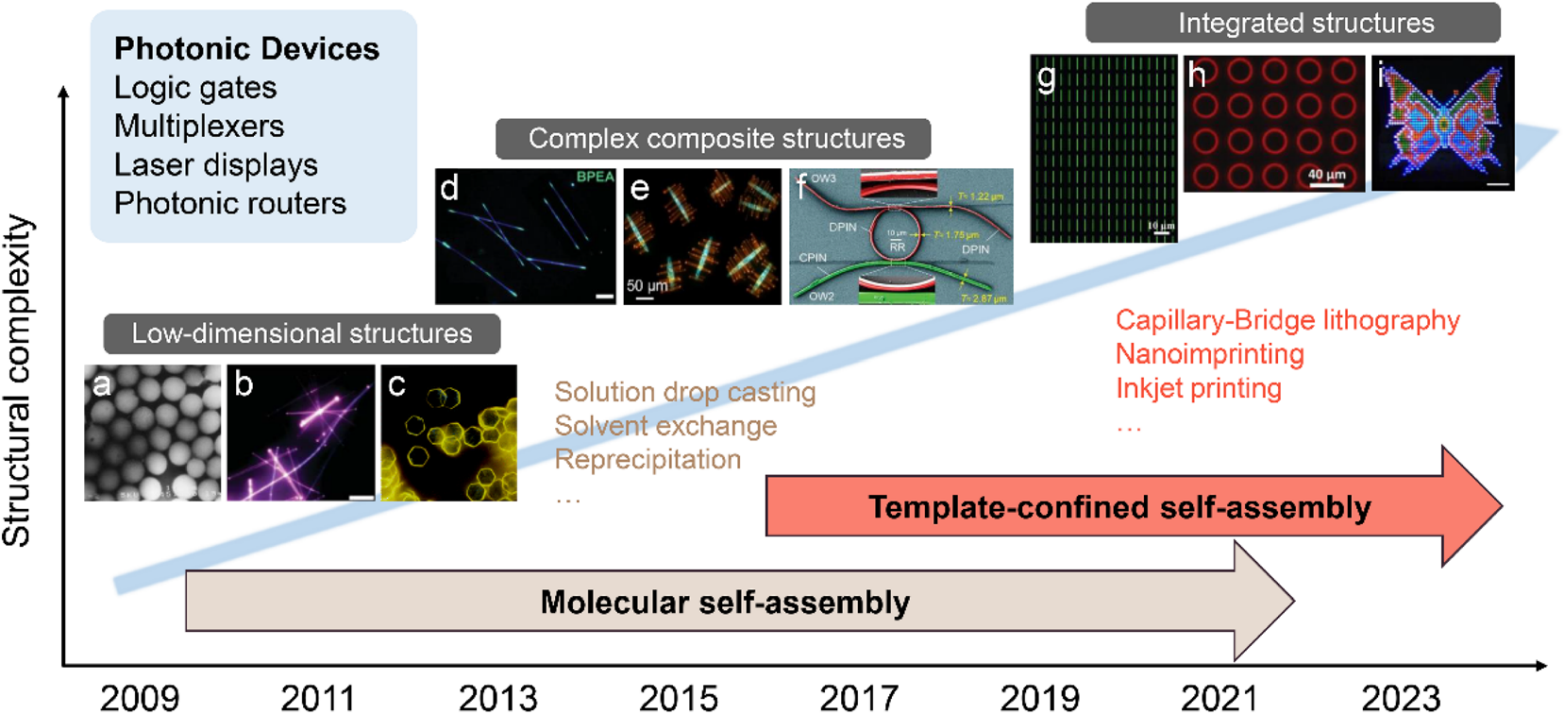
Schematic illustration of representative organic micro/nanostructures prepared by the molecular self-assembly strategy and the template-confined self-assembly strategy. Organic micro/nanostructures like low-dimensional structures, complex composite structures, and integrated structures have been widely applied in the photonic devices of logic gates, multiplexers, laser displays, and photonic routers. (a) Reprinted with permission from ref. 106. (b) Reprinted with permission from ref. 108. (c) Reprinted with permission from ref. 105. (d) Reprinted with permission from ref. 104. (e) Reprinted with permission from ref. 107. (f) Reprinted with permission from ref. 85. (g) Reprinted with permission from ref. 100. (h) Reprinted with permission from ref. 101. (i) Reprinted with permission from ref. 102.

Remarkable advancements in organic photonics are facilitated by the controllable preparation of organic nanomaterials with specific photonic properties, which are important prerequisites for understanding light–matter interactions and fabricating photonic building blocks at the micro/nanoscale. The photonic properties of organic nanomaterials are not only determined by the chemical structure of the constituent molecules but also highly rely on their shape, size, and surface structure.^26–28^ For example, one-dimensional (1D) structures with field confinement in two dimensions can serve as a Fabry–Perot (F–P) resonator,^29,30^ while two-dimensional (2D) structures that confine photons in one dimension have been demonstrated to serve as a whispering gallery mode (WGM) resonator.^31^ Thus, controlling the structure of organic nanomaterials offers a potent instrument for obtaining desirable photonic functions. In fact, many attempts to design and modulate the structure of organic nanomaterials have been made by scientists worldwide,^32–35^ especially after the successful reporting of molecular self-assembly strategies driven by weak intermolecular interactions (including van der Waals forces, π–π interactions, hydrogen bonds, and CH–π interactions). In the early stages, our research group and other research groups demonstrated abundant low-dimensional micro/nanocrystals^36–39^ (*e.g.*, 0D nanoparticles, 1D nanowires, nanotubes, and 2D nanosheets) prepared by reprecipitation,^40,41^ solution drop-casting,^42,43^ solvent evaporation method,^44,45^*etc.*, which provide novel device structures and resonator types for organic photonic devices (Fig. 1a–c). The structurally well-defined micro/nanocrystals exhibit specific optoelectronic properties, high optical cross-section, low trap density, and good mechanical flexibility. These unique characteristics enable them to serve as fundamental building blocks for various light-based functionalities, including light generation, propagation, modulation, and detection.^46–48^

With the increasing understanding of molecular self-assembly, research on the self-assembly of organic molecules has gradually expanded to the preparation of more complex composite nanomaterials for advanced practical functions of organic photonic devices. For example, by properly designing the intermolecular interactions between different organic compounds and optimizing the preparation conditions (temperature, concentration, solvent properties, *etc.*), the cooperative assembly behaviours can be controlled, thereby forming complex composite nanomaterials.^49–53^ However, the weak intermolecular interactions cannot to coordinate multiple building blocks across different length scales, resulting in the assembly of multiple components preferring to generate a homogeneously mixed structure or to undergo phase segregation in most cases. To further enhance the control over the molecular self-assembly process, several novel construction strategies assisted by external driving forces have been developed, including localized epitaxial growth,^54,55^ template-guided combination,^56^ and micromanipulation-assisted integration^57^ (Fig. 1d and e). These strategies allow the assembly of a wide range of simple low-dimensional nanomaterials into complex heterostructures with high spatial and angular accuracy, such as 1D axial heterostructures,^58–60^ dendritic heterostructures,^61^ embedded heterostructures,^62^ core/sheath heterostructures,^63–65^ and multi-bent junctions.^66,67^ Rational coupling of low-dimensional nanostructures into heterostructures not only effectively preserves their respective features, but also generates novel photonic properties due to charge and energy transfer at the coupling interface.^68,69^ Such complex heterostructures play a critical role in the development of photonic circuits, such as photonic routers,^70–73^ photonic modulators,^74–76^ logic gates,^77,78^ wavelength filters,^79^ and multiplexers.^80,81^ Furthermore, significant progress in the field of mechanophotonics,^82–86^ which integrates flexible molecular crystals and atomic force microscopy tip-based mechanical micromanipulation, has propelled the advancement of complex photonic structures and enabled the realization of photonic integrated circuits with enhanced functionality (Fig. 1f).

In addition to a high level of morphology control, precise control of the orientation and position of organic low-dimensional nanostructures on surfaces is important for the predictable and controllable integration of photonic devices. The fabrication of organic nanostructure arrays with specific morphology can therefore enable the wide application of integrated devices, including laser display, information coding, and sensing.^87–91^ However, organic nanostructures obtained by molecular self-assembly in solution often have a random orientation and position and are not suitable for simultaneous integrated processing. Thus, the integrated processing of organic nanostructures places higher demands on the control ability during the self-assembly process. The “template-confined self-assembly strategies” allow confining the precursor solution in micro-/nano-level restricted spaces to control the morphology and deposition location of the nanostructure, which has the advantages of high controllability and structural stability.^92,93^ This technique enables the simultaneous growth and patterning of organic molecules, resulting in the production of diverse patterned organic micro/nanostructures.^94–99^ L. Jiang *et al.* developed a versatile “capillary-bridge lithography” technique to pattern 1D single crystal arrays by manipulating the generation and dewetting of microfluids (Fig. 1g).^100^ WGM microring arrays have been successfully prepared by soft lithography, and their laser properties have also been intensively discussed by H. Fu *et al.* (Fig. 1h).^101^ As shown in Fig. 1i, the multicolor microlaser arrays have been prepared through inkjet printing, and their application in full-color laser displays has been demonstrated by Y. S. Zhao *et al.*^102^

The development of assembly strategies assisted by external driving forces has enabled controlled preparation from simple low-dimensional structures to hierarchical structures, including homo/heterostructures and micro/nanostructure arrays.^103–108^ Based on the increased complexity and orderliness of organic nanomaterials, a wealth of integrated photonic devices have also been successfully developed. It follows that exploring an efficient external driving force in the assembly process is critical for organic photonics to move from basic research to practical applications. Recently, our group and other research groups have found that using a magnetic field could be an elegant way to prepare organic hierarchical nanostructures, benefiting from the controllable arrangement and precise positioning of organic low-dimensional structures.^109–111^ Despite being in its nascent stages, magnetically controlled assembly methods in the preparation of organic hierarchical nanostructures provide some distinct features: (i) the high controllability of the magnetic field allows for the on-demand integration of multiple components by accurately manipulating their spatial position, direction, and order.^112,113^ Such integration of diverse components can produce many novel photonic functionalities. (ii) Magnetic field-induced driving forces can enhance the interactions between components or compete with them to produce a new equilibrium state for driving the assembly process.^114^ This equilibrium state can be dynamically adjusted by active fine-tuning of the field-induced driving forces, thus enabling the structural and temporal programming control of hierarchical nanostructures.^115,116^ (iii) Various forms of magnetic fields, such as uniform, nonuniform, rotating, and alternating fields, can be used to perform different types of operations on organic nanomaterials, such as mixing, capturing, transporting, and rotating. At the same time, more precise and flexible magnetic control systems have been developed, such as micromagnets and microcoil arrays.^117^ Overall, the magnetically controlled assembly of organic nanomaterials is a promising technology that offers us a new approach to achieving organic integrated photonic devices with previously unthinkable structures and functions.

Although several excellent reviews/perspectives related to magnetically controlled assembly/manipulation have been reported,^118–121^ most of them focused on magnetic nanomaterials or materials without photonic properties. Therefore, a perspective that can provide the application of magnetically controlled assembly strategies in the preparation of organic hierarchical nanostructures, including homo/heterostructures and microstructure arrays, is in high demand for the pursuit of organic integrated photonics. From this perspective, we aim to offer an overview of recent advances in magnetically controlled assembly in organic photonics from the perspectives of fundamentals, assembly methods, and applications. We begin with an introduction to the principles and fundamentals of the magnetically controlled assembly method. Next, we provide an overview of the latest advances in magnetically controlled assembly methods, such as magnetic interparticle interaction control assembly, localized gradient magnetic field control assembly, magnetic potential trap control assembly, and dynamic magnetic potential trap control assembly. Moving forward, three representative applications, microlasers, laser displays, and information encryption, are analyzed in detail. Finally, we present perspectives on the future opportunities and challenges of magnetically controlled assembly strategies in the field of organic integrated photonics.

## 2. Fundamentals and principles

### 2.1 Magnetically controlled assembly strategy

**Fig. 2:**
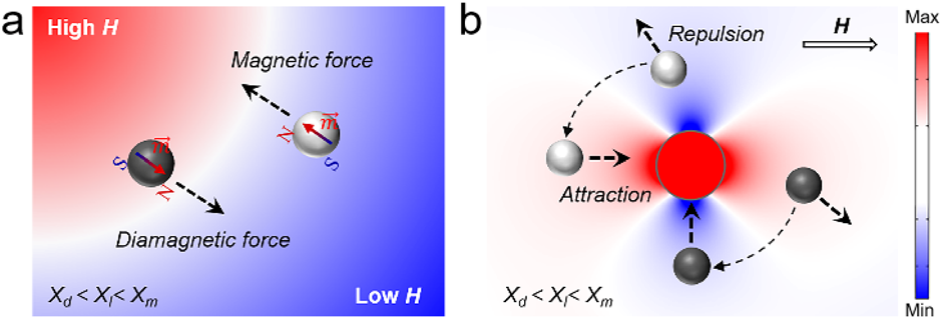
(a) Schematic illustration of positive magnetophoresis and negative magnetophoresis. A magnetic object (white sphere) levitated in the liquid medium (*χ*_m_ > *χ*_l_) experiences a magnetic force, moving towards an area of the field maximum; a diamagnetic object (black sphere, *χ*_d_ < *χ*_l_) experiences a diamagnetic force, moving towards an area of the field minimum. (b) Schematic illustration of the assembly behaviour of a magnetic object and a diamagnetic object under a gradient magnetic field.

As early as the beginning of the 20th century, scientists began to study the rules of magnetic objects' motion in a magnetic field and found that magnetic objects move along the direction of magnetic force lines, laying the foundation for the later development of magnetic field-based manipulation technology.^122^ Under an inhomogeneous magnetic field, the magnetic force *F*_m_ acting on a magnetic object can be expressed as follows:^123^1
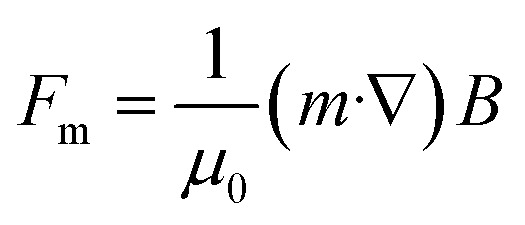
*μ*_0_ represents the vacuum permeability, *m* represents the magnetic moment, and *B* represents the total magnetic induction. According to eqn (1), it is necessary to provide a magnetic field gradient to create a magnetic force applied to a magnetic object. One of the most direct ways to establish a field gradient is to use different types of magnets, including permanent magnets, electromagnets, and other novel magnetic field control systems, which can directly produce and regulate a gradient magnetic field. When a magnetic field gradient is applied to the magnetic object, a dipole-field force (*i.e.*, packing force) can be induced to drive the object to move in the direction of the field gradient toward strong field regions, termed positive magnetophoresis (Fig. 2a). Further analysis of the magnetostatic potential energy *E* of the magnetic object under an inhomogeneous magnetic field allows a better understanding of this magnetic response, which can be mathematically described as:2
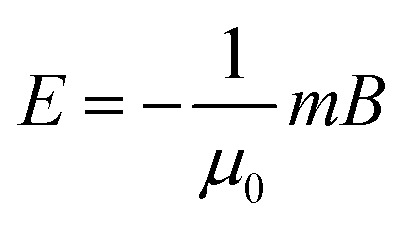


Eqn (2) shows that the potential energy is negative for magnetic objects with magnetic dipole moments (superparamagnetic or ferromagnetic objects). Hence, the magnetic objects move towards regions of maximum field strength to achieve greater stability by minimizing their potential energy. Based on this principle, a magnetically controlled assembly strategy suitable for magnetic nanostructures was exploited. Under the action of an external magnetic field, the magnetic moments of the magnetic nanostructures tend to be aligned in the same direction as the magnetic field, so that a local magnetic field is formed around them. Due to the nonuniform distribution of the magnetic moment on the surface of the nanostructures, the field direction and strength acting on different positions of the magnetic moment are different. As a result, the magnetic nanostructures also generate a gradient magnetic field on their surface. This gradient magnetic field can be used to manipulate the motion and positioning of adjacent magnetic nanostructures *via* positive magnetophoresis.

The self-assembly process is a competition between the various interactions acting on the nanostructures and the process of minimizing the energy of the system. For magnetic nanostructures, there are usually several possible interactions: van der Waals attraction, magnetic dipole attraction, and electrostatic repulsion. Magnetic field-induced packing forces do not directly interfere with the interactions between the magnetic nanostructures, but they can enhance or compete with them to produce a new equilibrium state for driving the assembly process. If the magnetic nanostructures are sufficiently separated from each other, the magnetic dipole–dipole interactions between them are negligible. Therefore, the packing forces generated by the coupling of the induced dipole and the applied field dominate their magnetic response behaviour.^124^ The effect of the packing force on the assembly behaviour of nanostructures depends on the type of applied field and the magnetic anisotropy of the nanostructures. In a uniform applied field, the magnetic field-induced packing force causes magnetic nanostructures with anisotropic magnetic properties to generate a magnetic torque; while in an inhomogeneous magnetic field, the packing force can drive the magnetic nanostructures to move along the magnetic field gradient and form aggregates. When two magnetic nanostructures become closer, the magnetic dipole–dipole interaction becomes critical in controlling their motion and assembly behaviour (as illustrated in Fig. 2b). At this point, the magnetic dipole–dipole interaction determines the bonding configuration and spatial order of the nanostructures and therefore affects the physical and chemical properties of the final assemblies. It should be noted that the magnetic dipole–dipole interaction is one type of magnetic gradient force.

In the 2004s, B. Hou *et al.* used a weak magnetic field to achieve the self-assembly of single-crystalline Fe_3_O_4_ nanowires.^125^ Subsequent research findings have provided further evidence that a magnetic field can effectively promote the formation of magnetic nanomaterials with unique structures.^126,127^ To date, magnetic fields have successfully guided a variety of magnetic nanoparticles to form ordered structures, such as nanochains, nanobelts, nanosheets, and bulk structures.^128–131^ It is worth noting that magnetically controlled assembly is often a non-contact and template-free method that does not cause damage to the resulting nanomaterials, and therefore results in superior photonic and electronic properties. More interestingly, the equilibrium state between particles can be dynamically adjusted by active fine-tuning of the field-induced packing force, thus enabling the structural and temporal programming control of assembled nanomaterials.^132–135^ The magnetic field has undoubtedly been recognized as an essential controllable external force due to its numerous advantages in the controllable fabrication of structural nanomaterials, including precise assembly and dynamic assembly.

### 2.2 Magneto-Archimedes effect

Most organic photonic materials, including organic small molecules and polymers, are diamagnetic, and their magnetic susceptibilities are extremely low, typically ranging from 10^−8^–10^−4^.^136^ Thus, the magnetically controlled assembly of organic photonic materials is hindered by the fact that diamagnetic objects exhibit an almost negligible net magnetic force *F*_m_ when exposed to a gradient magnetic field. The history of manipulating the motion and assembly of diamagnetic objects using magnetic fields can be traced back to the late 20th century when diamagnetic objects such as graphite and bismuth were first suspended in the air *via* a non-uniform magnetic field.^137,138^ In the early 21st century, a more efficient and stable diamagnetic levitation was developed using the perfect diamagnetic effect of superconductors in strong magnetic fields.^139–142^ Subsequently, a manipulation technology based on the “magnetic Archimedes effect” was developed, providing a new idea and technical support for manipulating the motion and assembly of diamagnetic objects using magnetic fields.^143,144^

Under an inhomogeneous magnetic field, the magnetic force *F*_m_ acting on a diamagnetic object placed in a liquid medium can be expressed as follows:^145,146^3
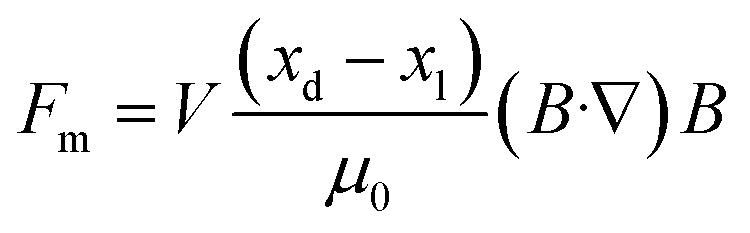
*χ*_d_ and *χ*_l_ represent the magnetic susceptibility of the diamagnetic object and the liquid medium, respectively. *V* represents the object's volume. Note that to simplify the calculation, the object is approximated as spherical particles whose dipole moment can be considered equivalent to a point dipole. The “magnetic Archimedes effect” differs from positive magnetophoresis because it takes advantage of the purposely increased contrast in magnetic susceptibility (*χ*_d_ − *χ*_l_) among the target object and its surrounding environment. When a diamagnetic object (*χ*_d_ < 0) is placed in a paramagnetic fluid (*χ*_l_ > 0), the object will move towards the regions of minimum field strength due to the negative difference in their magnetic susceptibility (*χ*_d_ − *χ*_l_), this magnetic response is also known as negative magnetophoresis (Fig. 2a). It is worth noting that the magneto-Archimedes effect allows for the manipulation of objects with lower magnetism than the surrounding medium, not just limited to diamagnetic materials. Such an effect effectively lowers the demand for extremely high magnetic fields. It increases the feasibility of magnetic field manipulation of organic materials, leading to a breakthrough in applying magnetic fields in the manipulation and assembly of organic materials from theory to practical application.

**Fig. 3:**
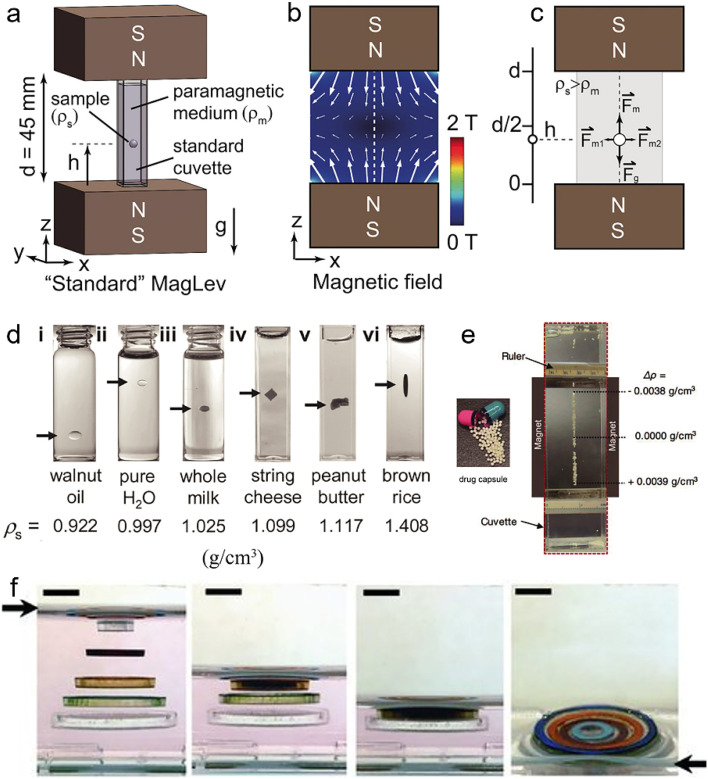
(a) The magnetic levitation system with two permanent magnets and a sample in paramagnetic medium. (b) Magnetic field produced by two identical magnets with the same poles aligned with each other. The dashed line indicates the central axis of the magnetic field used to levitate the object. (c) When the magnetic force, gravitational force and buoyancy force of an object are balanced, it reaches a stable suspension height. (d) Density characterization of various substances suspended in paramagnetic medium. (e) Identification and separation of mixed drugs suspended in paramagnetic medium. (f) A solid circular base and four plastic rings self-assembled into a co-planar structure after removing the suspension medium. (a–c) Reprinted with permission from ref. 150. (d) Reprinted with permission from ref. 151. (e) Reprinted with permission from ref. 152. (f) Reprinted with permission from ref. 153.

A variety of manipulation and levitation strategies based on the magneto-Archimedes effect have been developed for adaptation to organic objects and have broad application prospects in characterizing materials,^147^ assembling objects,^148^ bioanalyses,^149^ and many others. Magnetic manipulation/levitation tools commonly comprise two permanent magnets in which the same poles are aligned with each other to produce a larger magnetic field gradient (Fig. 3a and b).^150^ A container filled with a paramagnetic medium (*e.g.*, ferrofluid, an aqueous solution of MnCl_2_, CuSO_4_, FeCl_2_, or GdCl_3_) is placed between the magnets to produce a negative difference (*χ*_d_ − *χ*_l_) in the magnetic susceptibility between the object and surrounding liquid. For an object submerged in the liquid, both gravitational forces and buoyancy forces are constant, but they do not balance each other most of the time. Therefore, an additional force is required to assist the object in achieving stable suspension in the fluid. As illustrated in Fig. 3b, a diamagnetic object at different locations is subjected to diamagnetic forces (*F*_m_) of different directions and strengths, provided by the magnetic levitation apparatus. The diamagnetic object needs to adjust its levitation height until the diamagnetic forces equilibrate with the resultant force (*F*_g_) of gravitational forces and buoyancy forces. Thus, the diamagnetic object is eventually able to levitate stably in a paramagnetic medium and the suspension height is determined by their densities (Fig. 3c).

G. M. Whitesides *et al.* applied magnetic levitation in density tests of matter. Fig. 3d shows distinct kinds of matter (such as oil, milk and foods) levitated in an aqueous GdCl_3_/CaCl_2_, illustrating that variations in levitation height among these matters were proportionate to differences in their densities.^151^ Based on the differences in suspension heights of matters with different densities, the simultaneous separation and isolation of the mixture of drugs can also be easily achieved using this method (Fig. 3e).^152^ F. Ilievski *et al.* demonstrated the application of magnetic levitation for templated 3D self-assembly.^153^ Adjusting the magnetic and gravitational forces acting on the building blocks allowed adjustment of their suspension height and spatial order. When the magnetic and gravitational forces of each building block are balanced, multiple building blocks in the paramagnetic medium can be arranged in an orderly manner into a layered structure. The suspended building blocks are stacked once the paramagnetic liquid is removed, enabling 3D self-assembly (Fig. 3f).

## 3. Magnetic controlled assembly for organic hierarchical structures

Organic hierarchical structures are formed by integrating multiple low-dimensional structures into a homo/heterostructure or an array structure and are emerging as an important class of functional microscopic architectures.^154^ Great demands have been placed on the innovation of synthetic strategies for efficient preparation with precisely controlled structures, sizes, and compositions of hierarchical structures.^155–157^ Very recently, our research group, together with other groups worldwide, has accomplished a series of studies on this subject. It has been shown that a magnetic field, as a non-contact and damage-free means, has been used to transport and assemble particles by introducing a new driving force on microscopic components, exhibiting unique capabilities in creating complex static structures as well as field-manipulated active structures.^158–160^ The unique advantages of magnetically controlled assembly methods enable scientists to design structures and functions on integrated photonic devices that were previously unattainable. In this section, we introduce several typical types of magnetically controlled assembly strategies.

### 3.1 Magnetic interparticle interaction control assembly

In magnetic fields, chain-like coupled structures induced by magnetic dipole–dipole interactions have been extensively studied in theoretical and experimental research.^161,162^ In the previous section, we investigated the formation mechanism of such chain-like structures by analyzing the interaction forces/energies of magnetic particles. Like magnetic particles, diamagnetic particles tend to assemble into chain-like structures along the magnetic field direction. This tendency has become a reality by adjusting the magnetization difference between diamagnetic particles and the medium, which can be easily understood using eqn (3). In this field, several studies have successfully predicted the assembly behaviour of diamagnetic particles in the presence of a uniform magnetic field.^163–165^ The magnetic force between two diamagnetic particles in a magnetic fluid can either attract or repel, depending on the angle between the applied field direction and the line connecting the particles' centres. The critical angle for spherical diamagnetic particles is approximately 54.7°, so the assembly structure can be adjusted *in situ* by dynamically adjusting the magnetic field direction.^166^ This feature also makes the dynamic assembly of complex structures possible. Although we have studied and understood the critical role played by magnetic dipole–dipole interactions in diamagnetic particle coupling, the characteristics of the final assemblies are determined by a variety of factors, such as magnetic field strength, medium properties, and particle parameters.^167^ Therefore, further understanding of the efficient utilization of magnetic dipole–dipole interactions between diamagnetic particles and the rational optimization of the above factors leads to better control over the assembly structure of organic hierarchical nanostructures.

**Fig. 4:**
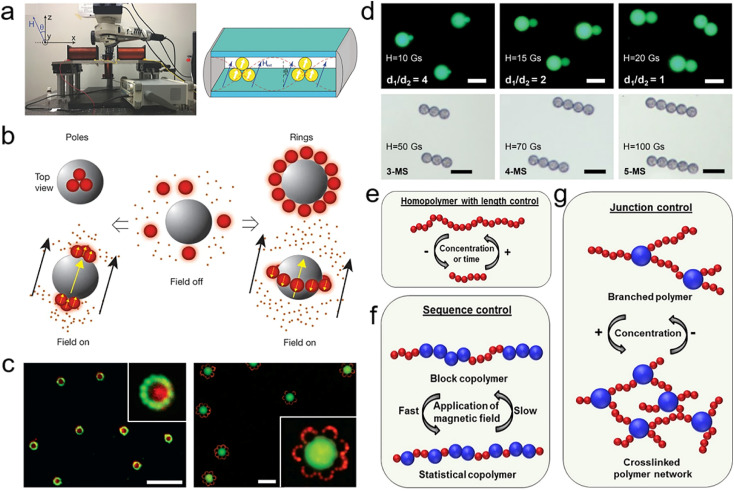
(a) Magnetically controlled assembly system with a magnet, a sample in a paramagnetic medium and a microscope. (b) Assembly behaviour of colloidal particles with different magnetic susceptibility under a magnetic field. (c) The “two tone” and “flower” structures are obtained by designing the magnetism and dimensions of the three components. (d) PL images of double-sphere coupled structures with different size ratios and multi-sphere chain structures. (e) Chain-like structures formed by monodisperse nanoparticles. (f) Different-sized particles assemble into colloidal block copolymers. (g) Crosslinked networks formed by a mixture of particles with large differences in size. (a) Reprinted with permission from ref. 172. (b and c) Reprinted with permission from ref. 173. (d) Reprinted with permission from ref. 174. (e–g) Reprinted with permission from ref. 175.

B. B. Yellen *et al.* have made remarkable contributions to exploring how to construct more complex colloidal coupled structures by exploiting magnetic interactions.^168–171^ Typically, magnetically controlled assembly is performed in a paramagnetic liquid medium to ensure the effective generation of the magneto-Archimedes effect and sufficient mobility of the particles to assemble (Fig. 4a).^172^ The assembly process is initiated and controlled by a magnetic field, such as permanent magnets, electromagnets, micromagnet arrays, and micro-coils arrays, which are then monitored and recorded *in situ* by an upright microscope. In their work, magnetic and non-magnetic particles suspended in a ferrofluid are assembled into four types of complex structures: “poles”, “rings”, “flowers” and “two tone”.^173^ Under a uniform applied field, the magnetic moment of the magnetic particles tends to align with the field direction. A weak field region is formed in the plane surrounding the particles in a direction perpendicular to the applied field, where the magnetic fields generated by the particles oppose the applied field. At the poles of the magnetic particles, a region of strong field is formed in which the magnetic field of the particles strengthens the external field (Fig. 4b). Thus, through the positive/negative magnetophoresis effect, paramagnetic particles with magnetization greater than that of ferrofluid and diamagnetic particles with magnetization less than that of ferrofluid move to the strong and weak field regions, respectively. In this work, the size ratio of the particles and the concentration of the ferrofluid are important for the formation and modulation of complex colloidal coupled structures. By manipulating these parameters, a more diverse range of colloidal coupled structures can be designed and constructed (Fig. 4c).

In addition to magnetic nanostructures, which have been extensively studied for constructing gradient magnetic fields, it has been demonstrated that non-magnetic nanostructures can also be used to construct these fields for assembling complex coupled structures. Based on this strategy, our group assembled polystyrene microspheres into complex coupled structures in a more efficient and highly accurate manner. By carefully controlling the magnetic field strength and orientation, the size of the polystyrene microspheres, and the magnetic susceptibility of the liquid medium, among other factors, we can accurately specify the assembly of particles into a coupled double-sphere structure (Fig. 4d).^174^ Compared with the naturally occurring assembly method, the magnetically controlled assembly method can improve reproducibility and reliability, and the average yield of coupled structures can reach 80% under the optimized experimental conditions. Furthermore, we can obtain chain-like structures consisting of multiple microspheres by increasing the strength of the applied field. The magnetic field holds these structures together in a non-contact manner, allowing for non-destructive coupling of the contact points that form a strong foundation for subsequent optoelectronic applications. Building upon the discussion in Fig. 4d, it is possible to leverage magnetic fields further to control the formation of longer chain-like structures (Fig. 4e). As shown in Fig. 4f, polymer chains of a larger size are arranged selectively with smaller microspheres surrounding them, demonstrating that the magnetic field can effectively control the order of the selected particle sequence in the chain of microspheres.^175^ Furthermore, by introducing larger spheres, it is possible to control the formation of branching structures and create different two-dimensional assembled networks (Fig. 4g). In addition, magnetic fields can also be utilized to manipulate the assembly of anisotropic diamagnetic rod-like structures in various directions, forming diverse assembled structures.^176–178^

### 3.2 Localized gradient magnetic field control assembly

In the pursuit of building more sophisticated assembled structures, achieving precise control over the spatial positioning of the structures is becoming increasingly important.^179^ This is particularly true when working with micro/nanostructures, where even slight deviations in positioning can significantly affect the device's performance. One approach that has gained significant attention in recent years for achieving precise spatial control in assembled structures is magnetically controlled manipulation based on localized magnetic field gradients.^180^ By leveraging the unique properties of magnetic fields, it is possible to exert precise control over the spatial distribution of particles during assembly. This technique offers several advantages over other assembly methods, including a higher degree of controllability over the final structure and a greater degree of precision in placement.^181^

**Fig. 5:**
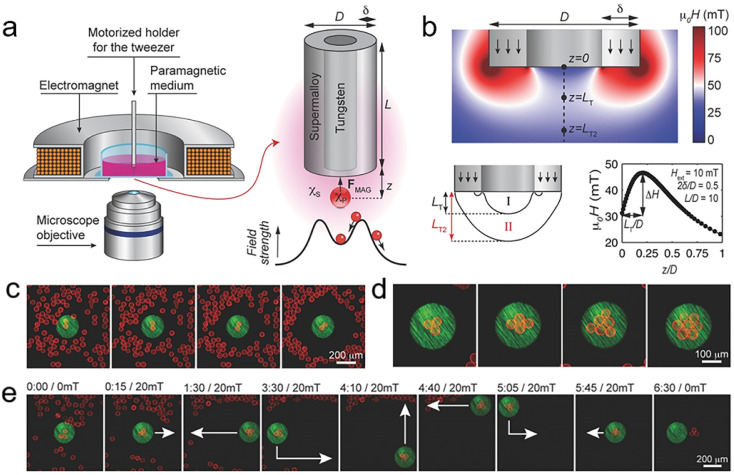
(a) Experimental setup and schematic illustration of particle trapping by a coaxial micro-pen. (b) Magnetic field distribution around the pen' tip. (c) Selective trapping of two silica beads. (d) Trapping of a larger bead cluster. (e) Image series of trapping, moving, and releasing a three-bead cluster. (a–e) Reprinted with permission from ref. 183.

To achieve controllable magnetic self-assembly at spatial operation points, the most straightforward problem one must face is how to construct and control the localized magnetic field gradients. B. A. Grzybowski and other researchers have devised a novel and sophisticated method that involves the preparation and application of miniature magnetic tweezers.^182^ These tweezers are designed to manipulate diamagnetic micro-objects in a paramagnetic medium and offer an exact and customizable approach to constructing complex coupled structures. These tweezers consist of two core components: an external electromagnetic coil and a miniature pen custom crafted for assembly and manipulation (Fig. 5a).^183^ The miniature pen features a weak magnetic tungsten core, which boasts a magnetic susceptibility of approximately 10^−5^ and is wrapped in a highly magnetic ferromagnetic material with a susceptibility of approximately 10^5^. This stark contrast in magnetic properties between the two materials enables the generation of highly localized and distinct magnetic fields around and within the pen in the presence of an external magnetic field, which forms the basis of their unique and innovative assembly approach (Fig. 5b). Upon activation by an applied field, strong fields emanate from both above and below the ferromagnetic casing of the pen, whereas a weaker field extends below the tungsten core, thereby creating a local gradient magnetic field that can attract or repel diamagnetic particles in proximity based on their precise locations. Leveraging this unique and dynamic movement of diamagnetic particles, magnetic tweezers can perform multiple functions, including selective and accurate manipulation of particle positions to create densely stacked structures at specific and release pre-determined locations, as shown in Fig. 5c–e.

### 3.3 Magnetic potential trap control assembly

Large-scale precise localization and patterning of organic micro/nanostructures is a prerequisite for their practical applications.^184,185^ The magnetic field as an external control system with the advantages of high controllability, non-contact operation and instantaneous response has excellent potential for precise manipulation and assembly, bringing new ideas for the patterned preparation of organic micro/nanostructures. It has been shown that introducing local gradient magnetic fields at specific spatial points in space can effectively restrict the spatial location of diamagnetic materials.^186,187^ Inspired by this, arranging multiple local gradient magnetic fields in specific positions and patterns may be able to rapidly organize and position diamagnetic organic micro/nanostructures. An easy and efficient way to generate local gradient magnetic fields with a specific arrangement is to introduce an array of micro/nano magnetic elements under an external uniform magnetic field.^188,189^

**Fig. 6:**
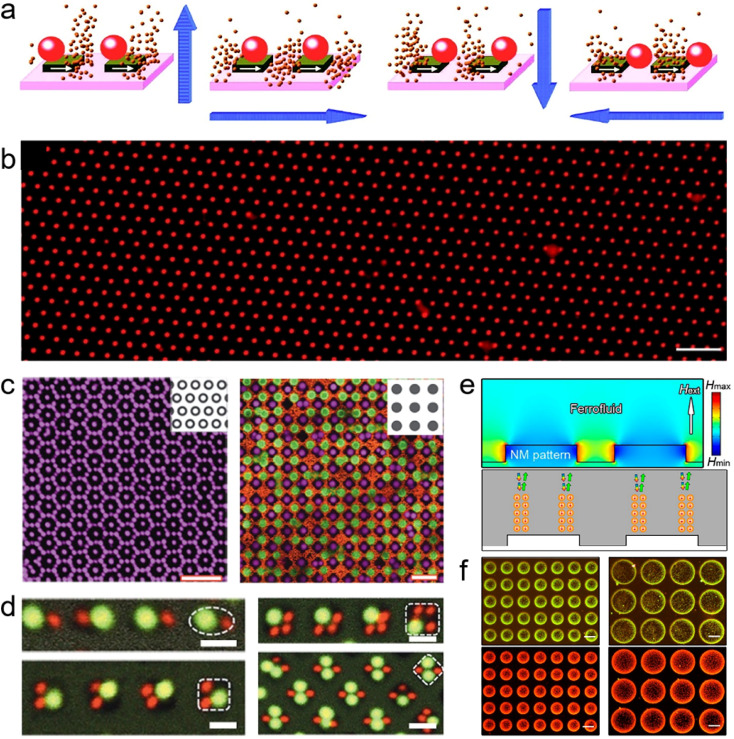
(a) Schematic illustration of magnetically controlled assembly of the diamagnetic particles on the array of magnetic potential traps. (b) Large single-layer arrays of colloidal particles prepared by constructing an array of magnetic potential traps. Various styles of assembly patterns prepared by using different nickel grids (c) and different configurations of mixed colloidal particles (d). (e) Simulated magnetic field distribution around the nonmagnetic pattern. (f) Various structured colour patterns formed by assembling microspheres of different sizes on the nonmagnetic pattern. (a and b) Reprinted with permission from ref. 190. (c and d) Reprinted with permission from ref. 191. (e and f) Reprinted with permission from ref. 193.

For example, G. Friedman *et al.* constructed a local gradient magnetic field with a two-dimensional arrangement for localizing and assembling colloidal particles in the ferrofluid.^190^ Magnetically controlled assembly is typically performed in the thin film (a uniform suspension of colloidal particles in the ferrofluid) between the cobalt microstructure substrate and the glass slide. Solenoid coils with iron cores are used to provide a homogeneous magnetic field (0–200 Gs) to manipulate colloidal particles. Fig. 6a shows that when a homogeneous magnetic field is applied to the cobalt microstructure, local low magnetic fields (usually labelled magnetic potential traps) appear where the cobalt microstructure's magnetic field subtracts from the applied field. For instance, when the applied field and the magnetization direction of the cobalt microstructure are aligned, the stray field on the surface of the microstructure and the applied field counteract one another, resulting in the formation of local low magnetic fields. As a result, diamagnetic colloidal particles in the ferrofluid are attracted to the local low magnetic fields at the top of the cobalt microstructure (Fig. 6b). In contrast, the opposite-direction magnetic field causes the local low magnetic fields to move to the space between two consecutive cobalt microstructures. In this circumstance, colloidal particles assemble on the other side of the cobalt array. The aggregation of colloidal particles on the substrate can be effectively avoided by creating a dynamic steady-state pattern of potential traps using a rotating homogeneous magnetic field. When the strength of the in-plane rotating field is greater than the coercivity of the cobalt microstructure (50–70 Gs), rotation of the magnetization of the cobalt microstructure can be induced. Therefore, the steady-state energy minimum is always located on the top of the cobalt microstructure, which results in the reliable packing of non-magnetic colloidal particles. More importantly, the colloidal particles located in other regions can be swept away by magnetic nanoparticles circulating around the cobalt microstructure, eventually forming a large single-layer array of colloidal particles.

Based on this strategy, B. A. Grzybowski *et al.* further explored the flexibility and versatility of magnetically controlled assembly methods in preparing large-area arrays of micro/nanostructures.^191^ By designing the arrangement of the nickel microstructure, a wide variety of magnetic-potential-trap patterns can be produced. Based on the positive and negative magnetophoretic effects of colloidal particles suspended in paramagnetic liquids (Ho(NO_3_)_3_ in DMSO/H_2_O), the diamagnetic and paramagnetic colloidal particles can aggregate inside and outside the potential traps, respectively (Fig. 6c). In addition to the positive/negative magnetophoretic effect, the aggregate behaviour of colloidal particles under the magnetic potential trap is also influenced by particle size. As shown in Fig. 6d, the magnetic potential trap at the top of the nickel microstructure will attract diamagnetic colloidal particles to aggregate on its surface, where large spheres with larger magnetophoretic forces will preferentially occupy the surface of the nickel microstructure. However, the top of a single nickel microstructure cannot hold two large spheres due to size limitations, so it can only continue to attract smaller spheres in the peripheral region. Therefore, not only AB_2_ or A_2_B_2_ configurations but even more complex configurations can be assembled by rational design of the particle size.

It is not only the magnetic microstructure array that can generate local gradient magnetic fields. Non-magnetic microstructure arrays can also modulate external magnetic fields with the assistance of magnetic fluids.^192^ Y. Yin *et al.* reported a versatile method for assembling non-magnetic objects at specific locations with high spatial resolution using a non-magnetic microstructure substrate.^193^ They constructed a raised microstructure array based on a non-magnetic polymer pattern and immersed it in a ferrofluid so that a minimum magnetic field appeared above the raised structure where a uniform magnetic field was applied (Fig. 6e). As a result, they successfully assembled diamagnetic microspheres on the array point with multiple spheres assembled on each array point, showing a densely packed arrangement. Moreover, the variation in the field parameters led to the formation of many 1D chains of microspheres on the raised structure, and these chains showed one-dimensional periodicity comparable to the wavelength of visible light, thus producing strong optical diffraction (Fig. 6f).

### 3.4 Dynamic magnetic potential trap control assembly

Dynamic assemblies are formed by dynamic interactions and non-covalent bonds with molecules or micro/nanostructures as building blocks.^194–196^ These materials are gaining recognition as a class of functional materials with stimuli-responsive, reversible, and self-healing properties. It covers a variety of applications ranging from catalysis, sensors, security and encryption, and optical displays to photonics.^197–199^ Building blocks can form static assemblies with specific structures under the balance of dynamic interactions and non-covalent bonds. Moreover, they can generate numerous intermediate configurations by actively adjusting dynamic interactions, providing increased functionality and adaptability to the environment.^200,201^ Therefore, introducing controllable dynamic interactions to precisely tune the assembly behaviours and dynamics of building blocks is crucial for generating materials that exhibit a broad range of functional and structural properties. Specifically, magnetic fields can induce directional magnetic dipolar interactions, facilitating precise regulation of the position and orientation of building blocks.^202^ Meanwhile, the high controllability of magnetic interactions in both time and space provides opportunities to design or dynamically adjust the structure and properties of the macroscopic assemblies.^203^

**Fig. 7:**
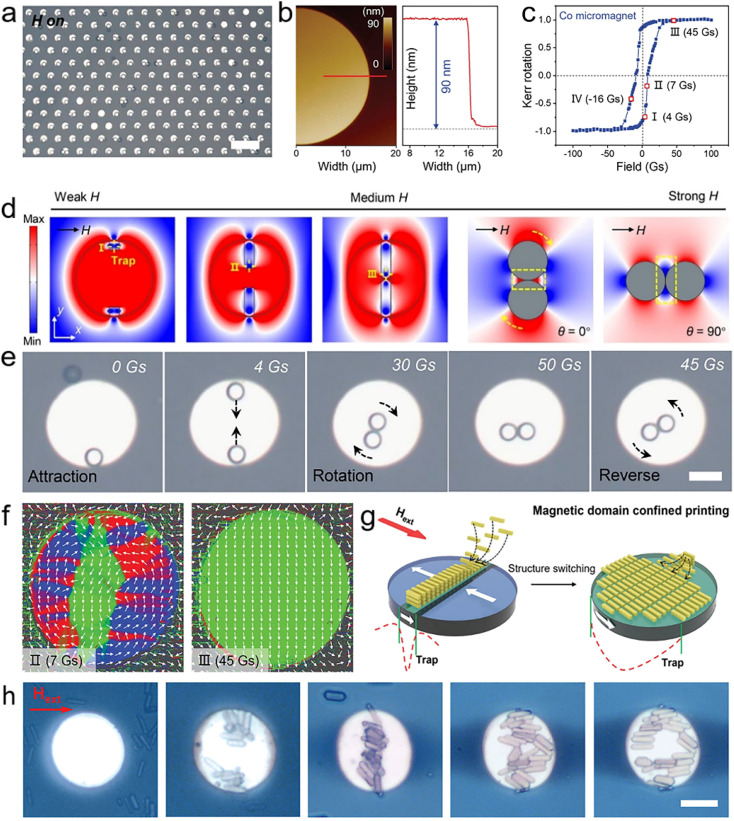
(a) A large-scale microspheres array is assembled on a micromagnet substrate. (b) AFM image and height diagram of individual micromagnet. (c) M–H loop of the micromagnet measured by longitudinal MOKE measurement. (d) Simulated field distribution of a micromagnet and a coupled microsphere. (e) Attraction and rotation process of a microsphere pair on a micromagnet under a magnetic field sequence. (a), (d and e) Reprinted with permission from ref. 204. (f) Plots of the magnetisation vector field on the micromagnet substrate obtained by quantitative Kerr microscopy. (g) Schematic illustration of magnetic domain confined printing method in which microrod aggregates switch shape in response to changes in the magnetic potential trap. (h) Macroscopic shape changes of microrod aggregate under a magnetic field sequence. (b, c) and (f–h) Reprinted with permission from ref. 205.

Recently, our group constructed large-scale patterns of magnetic potential traps generated by a ferromagnetic microstructure array to assemble polymeric microspheres suspended in ferrofluids (Fig. 7a).^204^ Benefiting from the size uniformity of the polymeric microspheres and ferromagnetic microstructure used in the assembly process, the control of the magnetic-potential-trap patterns over the states of the microsphere is highly consistent and reproducible. A magnetic hysteresis (M–H) loop of tiny regions on the surface of individual micromagnets was obtained by longitudinal magneto-optical Kerr effect (MOKE) measurements. The results showed that the switching of magnetization direction occurred gradually with increasing field strength instead of a sharp transition at the coercive field observed in bulk ferromagnets (Fig. 7b and c). This implies that the spatial distribution of the local magnetic field on the surface of the micromagnet can be controlled by altering the strength of the applied field. Simulations of the local field distribution demonstrate that magnetic potential traps emerge at the top/bottom edges of the micromagnet under weak magnetic fields. As the field strength increases, the traps move toward the centre of the micromagnet (Fig. 7d). Thus, the polymer microspheres in the ferrofluid are coupled along a specific trajectory under the action of the potential trap (Fig. 7e). With a rise in field strength, the magnetic torque of the coupled microspheres becomes more critical. In this situation, the entire coupled structure rotates and aligns its long-axis direction with the field direction as the field strength increases. The motion (attraction and rotation) of polymer microspheres under the applied field is highly reproducible (over 1000 cycles) with a fast temporal response and high spatial precision. This designable tuning of the motion paths of microspheres on individual micromagnets allows for the dynamic reconfiguration of the local arrangement and assembly configuration of the array units.

In the process of spontaneous magnetization of ferromagnetic materials, microscopic magnetization regions with different orientations, called magnetic domains, are formed inside to reduce the magnetostatic energy. If the size and shape of ferromagnetic materials and the external magnetic field change, the morphology of the magnetic domains evolves richly, so that the morphology of the magnetic potential trap formed at its top can be further controlled. Such magnetic potential traps generated by microscopic magnetic domains allow for more precise control of the assembly structure, and even tinier assembly units can be manipulated to form dynamic assemblies. Recently, our group developed a magnetic domain confinement assembly method that combines the evolution of the magnetic domain structure with a magnetically controlled self-assembly process.^205^ By using the magnetic domain pattern to provide a “virtual assembly template” for the assembly of organic microcrystals, the formation and change of the microcrystal aggregates array can perfectly replicate the random evolution of the magnetic domains. To visualize the evolution of magnetic domains in individual micromagnets, colour-coded images (Fig. 7f, performed by Dr I. Soldatov at the Leibniz Institute for Solid State and Materials Research) were obtained using an advanced wide-field Kerr microscopy approach developed by I. V. Soldatov and R. Schäfer.^206–208^ Based on this state-of-art technique, the magnetization can be quantitatively measured by vectorial analysis of the domain structure, and the vectors in colour-coded images profile the surface magnetization of the in-plane magnetic domains.

In the individual micromagnets, the morphology and position of the magnetic domains depend on the strength of the applied field and evolve dynamically with changes in field strength. The distribution of the magnetic potential trap above the micromagnet consists of an applied field and a stray field above the domain structure, where the stray fields enhance or weaken the applied field depending on the magnetization direction of the domain. Specifically, the stray field above the reversed domain is in the opposite direction to the applied field, thus partially weakening the applied field, while the direction of the stray field above the remaining domain is consistent with that of the applied field, so the effective magnetic field is enhanced through field strength superposition. Therefore, the morphology and position of the potential trap will match the magnetic domain pattern. As a result, diamagnetic microrods aggregate above the magnetic domain pattern under the attraction of the magnetic potential trap. The shape of microrod assemblies is dynamically adjusted with the evolution of the magnetic domains (Fig. 7g and h). Moreover, such a magnetic domain pattern can guide the movement and rearrangement of large-scale microrod assemblies, with more than 20 000 assemblies over 1 cm^2^. The magnetic domain confined printing method is a viable method for producing intricate structures with microscopically ordered and macroscopically tuneable structures. It offers a novel organic hierarchical micro/nanomaterial for designing and constructing next-generation organic integrated photonic devices.

## 4. Organic integrated photonic applications

The development of photonic applications based on various organic nanostructures has been a great success. However, with the further evolution of photonic applications toward complexity and practicality, there is a need to integrate photonic devices with different functions, which is one of the crucial reasons organic hierarchical nanostructures have gained so much attention. The versatility of organic hierarchical nanostructures has been well illustrated by current research. They combine the individual functions of various components and generate new properties *via* the synergistic effects between multiple components. Thus, the controllable preparation of organic hierarchical nanostructures provides a platform for the design and development of integrated photonic devices to create diverse photonic properties and functionalities that individual structures cannot achieve alone.^209,210^

Benefiting from advances in magnetically controlled assembly methods, it is now possible to assemble a broad range of organic hierarchical nanostructures in a highly accurate and efficient manner. Such hierarchical nanostructures have higher complexity and better controllability, which offers more possibilities for the design and development of novel integrated photonic devices. The highly controllable and instantaneous response of the magnetic field also allows the preparation of hierarchical nanostructures with dynamically tuneable structures, which facilitates the further development of functionally reconfigurable integrated photonic devices. In this section, we will present various novel photonic applications developed using these hierarchical nanostructures, including microlasers, laser displays, and information encryption.

### 4.1 Microlasers and laser displays

Benefiting from their small size, low power consumption, high-quality factor, easy integration, and diverse applications, single-mode lasers are an essential component of future integrated and miniaturized photonic devices. Expanding the free space range (FSR) by reducing the cavity size until only one mode is present in the resonant cavity is a common strategy for obtaining single-mode lasers.^211,212^ However, shortening the cavity size inevitably results in a poorer optical gain and more significant cavity loss, thus reducing the output power of the lasers. Fortunately, mode control has been achieved in a system of optically coupled microcavities *via* the Vernier effect.^213,214^ The basic principle is that two or more microcavities are coupled to each other to form a resonant cavity system, with one cavity acting as a spectral filter for the resonant modes of the other cavities.^215^ When the significantly increased FSR exceeds the spectral width of the gain medium, single-mode lasing occurs. Currently, precise control of the coupling and frequency detuning of inorganic semiconductor coupled microcavities by advanced micromanipulation techniques (such as FIB milling, electron-beam lithography, and chemical ion etching) is an effective method for tuning the resonant modes.^216^ However, there is still a lack of organic semiconductor-coupled microcavities with controllable structural parameters, which essentially limits their application in organic integrated photonics.

**Fig. 8:**
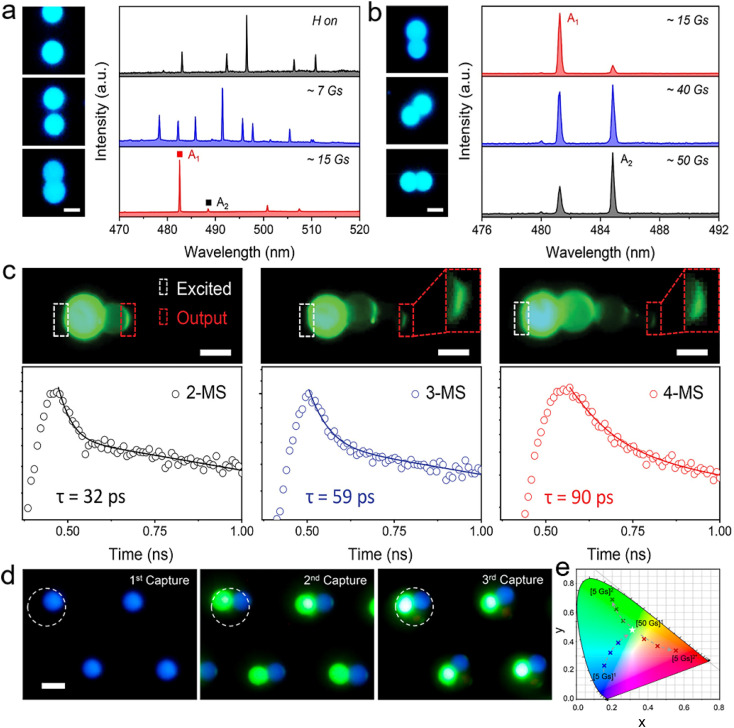
(a and b) PL images acquired from the attraction and rotation process of a microsphere pair and corresponding lasing spectra. (c) PL images of chain structures with different microsphere numbers and the corresponding decay curves of output light. (d) PL images of single-, double- and full-colour microsphere array. (e) CIE colour coordinates obtained in the structural reconstruction process of RGB pixels. (a, b) and (d, e)Reprinted with permission from ref. 204. (c) Reprinted with permission from ref. 174.

R. Chandrasekar *et al.* successfully fabricated polystyrene (PS) optical cavities with magnetically field-triggered orientation by incorporating magnetic iron oxide nanoparticles into polystyrene cavities.^217^ Doping dye molecules into the as-prepared microcavity can achieve luminescence of the whispering-gallery-mode, and the interference from doped magnetic particles on the luminescence can be neglected. Therefore, fluorescence spectra characterized by a *Q*-factor within the range of 400 were obtained, demonstrating the successful integration and functionality of the structured cavities. When an external magnetic field was further applied, the magnetic nanoparticles facilitated the collective alignment of polymer cavities, leading to the formation of coupled microsphere chains that were aligned along the direction of the magnetic field. Recently, our group also constructed coupled resonators with highly controllable structures that offer flexibility in tuning the optical resonance modes.^204^ Fluorescent dye-doped PS microspheres were first fabricated by the emulsion-solvent–evaporation method as high-quality whispering gallery mode (WGM) resonators. The coupled resonators were then obtained by coupling the pre-fabricated dye-doped PS microspheres on demand using a magnetically controlled assembly method. Benefiting from the precise and dynamic control capability of the magnetically controlled assembly method, the coupling distance and orientation of the coupled resonators can be adjusted over a wide range. As shown in Fig. 8a, the transition from multiple resonance modes to single resonance modes can be observed in the lasing spectra of the double-sphere coupled structure as they switch from the separated state to the coupled state. In such coupled structures, each spherical cavity serves as both a laser source and a mode filter, where overlapping resonant modes between microcavities are enhanced while other non-overlapping resonant modes are suppressed. This mode selection effect is strongly influenced by the orientation of the coupled resonators. Thus, the rotation of the double-sphere coupled structures under a magnetic field can be exploited to achieve polarisation switching of the lasing output. As shown in Fig. 8b, the lasing spectra of the double-sphere coupled structures with different rotation angles are collected under fixed vertical polarization, where two resonant modes compete with each other as the rotation angle changes. As a result, organic microlasers with tuneable resonance modes (including laser wavelength and polarisation) are successfully prepared.

The diversity and flexibility of the magnetically controlled assembly method provide ample opportunities for adjusting the structure of coupled resonators, such as the number and types of resonators in the coupled system. As shown in Fig. 8c, the number of microspheres in the coupled resonators can be further increased by increasing the magnetic field strength. As more microspheres are coupled, the lasing output becomes weaker due to the preferential nature of the high order during light propagation in a multi-sphere coupled structure.^174^ Such a multi-sphere coupled structure acts as coupled-resonator optical waveguides that can support the collective modes propagating back and forth within the coupled resonators, thus achieving an extended light propagation time.

Laser displays, with their inherent high monochromaticity and intensity of laser emissions, offer the potential for a significant shift in the display industry. To realize versatile and compact laser display panels, a dynamic modulation scheme for the optical parameters of laser pixels is now an urgent requirement. Recently, our group proposed a strategy that uses a magnetic field to externally control laser pixels, and it may take advantage of high controllability, non-contact properties, and instantaneous response. Full-colour coupled resonators can be produced by capturing three laser-dye-doped PS microspheres layer by layer (Fig. 8d). As shown in Fig. 8e, the full-colour coupled resonators present a chain structure at a field strength of 5 Gs, and only the microsphere at the center is excited by the laser spot fixed on the micromagnet, thus resulting in blue-colour lasing ([5 Gs]^1^). As the field strength increases, the full-colour coupled resonators are coupled at the center of the micromagnet, so that all three microspheres are integrally excited to obtain a full-colour lasing ([50 Gs]^1^). Due to the rotation process of coupled microspheres under a high field, the C153-doped microsphere initially located in the center of the chain structure was replaced by C6- or DCM-doped microspheres. Therefore, separate green- ([5 Gs]^2^) or red-colour lasing ([5 Gs]^2^*) was obtained during the decreasing-field process. This reconfigurable process allows the full-colour coupled resonators to achieve an extensive range of chromaticity as well as switchable colour expressions as an RGB pixel. The ability of the magnetic field to control the lasing output for the microlaser is critical to the development of laser display panels and other integrated photonic devices, which are expected to be further complementary to modulation by excitation, gain medium or substrate.^218^

### 4.2 Information encryption

The magnetic domain structure with a natural random distribution is often used to encode information for storage and transmission.^219,220^ This coding technology has very high security and anti-counterfeiting performance and is difficult to counterfeit and tamper with. However, the detection of magnetic domain encoding requires the use of special detection equipment and is relatively complex and costly to operate. The arrangement of luminescent materials in both vertical and horizontal sequences to form 2D luminescent patterns is regarded as a promising approach for developing optical recording devices and security tags. This is due to their ability to be rapidly read and their large storage capacity.^221^ The encoding and detection process of these luminescent patterns can significantly reduce the operational complexity of security applications. Thus, using magnetic domain structures to provide encoded information for 2D luminescent patterns combined with optical detection means reading the information quickly, providing another option for building high-security optical security.

**Fig. 9:**
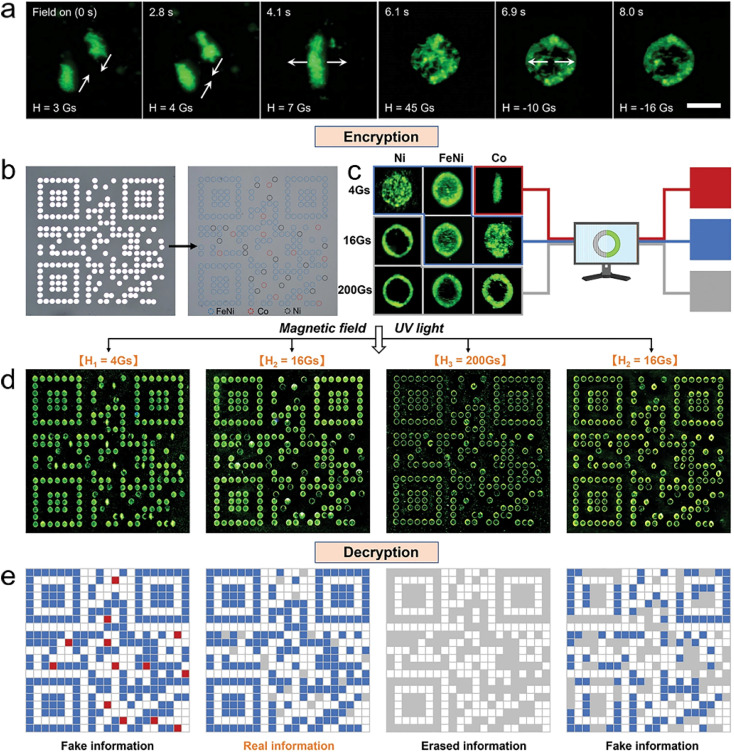
(a) Macroscopic shape changes of microcrystal aggregate under the applied fields. (b) Micromagnet array of a QR code covered by a nonmagnetic metal film. (c) Schematic illustration for the authentication of microcrystal aggregates as cryptographic primitives, where their shapes are recognized as red, blue, and grey squares, respectively. (d) Switching process of programmable microcrystal aggregated array under a magnetic field sequence of 4-16-200-16 Gs. (e) Corresponding QR codes are read by a custom-written image recognition program. (a–e) Reprinted with permission from ref. 205.

We have developed a magnetic domain confinement assembly method that combines the evolution of the magnetic domain structure with a magnetically controlled self-assembly process.^205^ By using the magnetic domain pattern to provide a “virtual assembly template” for the assembly of organic microcrystals, the formation and change of the microcrystal aggregates array can perfectly replicate the random evolution of the magnetic domains (Fig. 9a). First, different ferromagnetic metals are deposited at specific locations in the micromagnetic array to encode the magnetic domain information and manipulate the position and shape of the microcrystal aggregates (Fig. 9b). Multiple secure information can be expressed by reasonably designing the magnetic properties and arrangement of micromagnets. The geometry of the microcrystal aggregates can be used as secure information carriers. It can be easily recognized and encoded into a computer language by a customized image recognition program (Fig. 9c). Dynamic microcrystal aggregates exhibit unique properties in information encryption, including easy-to-identify encoding features, initial invisible features, high stability (∼100% reproducible), high-density encoding, and high integration density. Therefore, we fabricate a quick-response code for realizing the encryption, decryption, and erasure of multi-level security information by applying a specific magnetic field sequence (Fig. 9d and e). These results provide an in-depth understanding of the magnetically controlled assembly of microstructures as well as a new scheme for functional materials in high-security cryptography.

## 5. Conclusions and prospects

This perspective focuses on the development of organic hierarchical nanostructures based on magnetically controlled assembly methods, which are guided by the requirements of organic integrated photonics. Benefiting from the controllable arrangement and precise positioning of organic micro/nanostructures by a magnetic field, the prepared organic hierarchical nanostructures possess higher complexity and better controllability, which provides more possibilities for the design and development of novel integrated photonic devices. Meanwhile, field-manipulated active structures can also be prepared based on the high controllability and instantaneous response of the magnetic field, which facilitates the further development of functionally reconfigurable integrated photonic devices. Moreover, using magnetic fields to control particle–particle and particle–medium interactions can help reveal the assembly dynamics of organic micro/nanostructures. This approach enriches the traditional self-assembly theory by enabling a better understanding of collective particle behaviour. These basic principles and practical strategies have broad applicability and can be extended to other photonic materials, such as metal nanoparticles with surface plasmon resonance, and quantum dots with the quantum confinement effect. We believe that the increasing breakthroughs in magnetically controlled assembly technology will bring more vitality and opportunities for the research and development of organic photonic materials and promote their applications and development in integrated photonics.

As we enter the age of intelligence, the application scope of integrated photonics is growing and expanding. Currently, integrated photonics plays a crucial role in areas ranging from quantum communication, integrated sensors, and photonic computation to laser displays and wearables, and shows significant promise in material science research, precision measurement, and imaging. However, it is difficult for a single material or structure to meet all function-related needs of photonic devices. Hence, the quest for hierarchical self-assembly and high-quality heterogeneous integration has become a pivotal factor driving photonics' future growth. With ongoing advances in the adaptability and flexibility of photonic material integration, it is anticipated that the controlled preparation of abundant organic hierarchical nanostructures will contribute significantly to mitigating the current divide between electronic circuits and photonic circuits.

In recent years, magnetically controlled assembly methods have made exciting advances in the synthesis and assembly of organic hierarchical nanostructures. However, there are still many challenges to be addressed for further development: (i) the controllable magnetic force is the core tool for controlling the motion and assembly of micro/nanoobjects and needs to be designed and optimized to further improve the efficiency and precision of magnetically controlled assembly methods. On the other hand, new magnetic control systems need to be developed to attain specific magnetic field distributions and unconventional manipulation functions that exceed the potential of traditional magnets. In the future, as the demand for magnetic control systems continues to expand, it is crucial to meet additional requirements, such as controlling smaller objects and larger operating spaces. (ii) It is apparent from the research to date that the magnetic field-induced self-assembly of organic micro/nanostructures has produced many interesting phenomena and structures, and some subfields have matured to the point where they can be applied beyond academic research. To further advance this field, it is necessary to predict or design the desired structures according to the properties of the building blocks. Therefore, we need to enhance our comprehension of particle–particle interactions and self-assembly of particle ensembles through an external magnetic field and explore the basic mechanisms behind magnetically controlled assembly to guide the large-scale production of specific structured materials and promote the rational design of new manipulation methods. (iii) In addition to assembly strategies and theoretical research, the performance enhancement and functional expansion of organic hierarchical nanostructures are also important for expanding the practical applications of magnetically controlled assembly methods in the field of photonics. In particular, the dynamic response of hierarchical nanostructures under magnetic fields has generated new properties and dynamics, which demonstrate outstanding potential in the development of multifunctional and switchable photonic devices. Future research should explore how to translate these fascinating assembly phenomena and field-induced micro/nanostructures into useful multicomponent functional materials and optoelectronic applications, promoting their application and development in emerging fields.

Despite being a significant and highly anticipated challenge, we believe that the controlled preparation of organic hierarchical nanostructures through magnetically controlled assembly strategies will demonstrate the potential for new breakthroughs in the future.

## Author contributions

All authors contributed to the writing and revision of the manuscript.

## Conflicts of interest

There are no conflicts to declare.

## Supplementary Material
